# The dataset for the chronology of the sedimentation in the Danube abyssal fan which records the major episodes of the late-Holocene Black Sea evolution

**DOI:** 10.1016/j.dib.2022.108444

**Published:** 2022-07-07

**Authors:** Maria Ilie, Tiberiu Sava, Gabriela Cristea, Gabriel Ion, Dan Olteanu, Cristian Mănăilescu, Gabriela Sava

**Affiliations:** a RoAMS Laboratory, Horia Hulubei National Institute for R&D in Physics and Nuclear Engineering, Reactorului 30, Măgurele Bucharest 077125, Romania; b University of Bucharest, Faculty of Physics, Doctoral School of Physics, 405 Atomiștilor str. Ilfov, Măgurele 077125, Romania; c University of Bucharest, Faculty of Geography, N. Bălcescu, Bucharest, Romania; d Department of Mass Spectrometry, Chromatography and Applied Physics, National Institute for Research and Development of Isotopic and Molecular Technologies, Cluj-Napoca, Romania; e National Institute of Marine Geology and Geo-Ecology (GeoEcoMar), 23-25 Dimitrie Onciul St., Bucharest 024053, Romania

**Keywords:** Anoxic sediments, Sedimentation rate, Organic matter, Age-depth modelling, Black Sea

## Abstract

Anoxic marine sediments at the confluence with large rivers are key archives for monitoring the anthropogenic impact in the environment and asses the carbon sink character of oxygen deprived waters. This data article describes the analysis methodology and the results of the deep-sea sediments sampled from the NW part of the Black Sea, using the ^14^C dating, stable carbon isotopes, C/N ratio, metallic traces and ^210^Pb and ^137^Cs radioactivity. For this purpose, 26 sediment samples were taken from the MN183-3 sampling point (43.925.917 N, 30.758.911 E, 658 m water depth) using a Mark II-400-type multicorer. The samples were collected during the two weeks Mare Nigrum (MN) #183 marine expedition, which took place at the beginning of September 2018, in the Romanian section of the Black Sea shelf and continental slope. These analyses were employed in the construction of a Bayesian high-resolution sedimentation model, reported in M.Ilie et al. (2022).

## Specifications Table

**Table:** 

Subject	Stratigraphy
Specific subject area	The geochemistry and sedimentation rates for the NW Black Sea sediments;
Type of data	Tables, protocols
How the data were acquired	The ^14^C content measurement was determined using the 1 MV Tandetron® AMS system (HVEE, Netherlands) at Horia Hulubei - National Institute for R&D in Physics and Nuclear Engineering (IFIN-HH) in Bucharest; The organic sediment samples were pretreated for removal of the inorganic fraction and then converted to graphite using an AGEIII graphitization system (IonPlus AG, Switzerland) [11]. The sample combustion took place in an elemental analyser (EA) (Vario MicroCube, Elementar, Germany) connected to the AGEIII machine; The radiocarbon age calculation was made according to convention of radiocarbon age delivery [9], using the BATS software tool [10];The δ^13^C was measured using a Delta V Advantage stable isotope ratio mass spectrometer (Thermo-Fisher Scientific, MA, USA), while the C/N atomic ratios were provided by the EA; The measurements of the carbon species were identified on Primacs analyser from Skalar, The Netherlands; The X-ray fluorescence analysis were performed using the Bruker Tracer S1 Titan Spectrometer; ^210^Pb and ^137^Cs specific activities were measured using a low background gamma spectrometer with a HPGe semiconductor detector, 30% relative efficiency, model Ortec GMX-30-70-S, equipped with a 0.5 mm Be window.
Data format	RawAnalyzedFiltered
Description of data collection	For ^14^C dating the data obtained were normalized to modern radiocarbon level using Oxalic Acid II (NIST SRM 4990C), while the blank level was estimated using old charcoal of unknown origin. Additionally, secondary standards, i.e., IAEA-C5 and IAEA-C7 were also used. The data obtained for the carbon species were also normalized using Oxalic Acid II and IAEA-C1. The isotope ratio mass-spectrometer was calibrated against IAEA references material, NBS-22 oil. The elemental analyzer was calibrated using acetanilide C_8_H_9_NO, while the XRF spectrometer was calibrated using NIST 1646a standard [6].
Data source location	The sampling point is described by the following coordinates 43.925.917 N, 30.758.911 E, being located on the north-west continental slope of the Black Sea.
Data accessibility	Repository Name: Mendeley DataData identification number: https://doi.org/10.17632/4b8vhb45z2.2Direct URL to data: https://data.mendeley.com/drafts/4b8vhb45z2
Related research article	Maria Ilie, Tiberiu Sava, Alfred Vespremeanu-Stroe, Octavian Duliu, Gabriela Cristea, Gabriel Ion, Dan Olteanu, Cristian Mănăilescu, Gabriela Sava, A detailed chronology of the sedimentation in the Danube abyssal fan records the major episodes of the Late-Holocene Black Sea evolution, Quaternary Geochronology, Vol 70, 2022, https://doi.org/10.1016/j.quageo.2022.101279.

## Value of the Data

• A high-resolution sedimentation curve for the shelf and continental slope of the NW Black Sea region, based on ^14^C ages obtained on bulk organic matter (OM) preserved in anoxic conditions is notably desirable and quite unique for this geographic region. The anoxic/euxinic environments which are known to exhibit a preferentially conservative character for the OM are creating the perfect sedimentary archives. However, the quantitative estimations for the rate of carbon preservation are still missing from the literature, partly due to the lack of a high-resolution chronology. In this regard the present data play a major role in establishing the age-depth model on which one can further append information of interest such as total organic carbon (TOC), inorganic carbon (IC), molecular and other various markers of the organic matter provenance, in an attempt to understand the carbon-sink character of the Black Sea bottom waters.
• The researches undertaken within the field of sedimentation geochemistry with multiple applications such as global climate changes, oceanography and geoarchaeology will benefit from a high-resolution age-depth model which can be generated using various codes and software packages. Also, the excellent location of the sampling point makes these data remarkably valuable. The addressed area is a region of increased human activity and population dynamics during the prehistory and history, at the confluence of the long traveling Danube waters and the conservative anoxic environment of the Black Sea.
• Further insights can be developed in order to create new carbon accumulation models in this specific aquatic carbon sink where oxygen deprivation creates the perfect ground for estimating the carbon accumulation rates in marine sediments.

## 1 Data Description

Here, we publish the final data of our case study and intermediate products, which come in handy for creating a high-resolution sedimentation model spanning the last 5500 years.

The dataset available on Mendeley Data is composed of six Excel files, one pdf file and two zip files. Each Excel files is uniquely identified by the name encoded in the format: <Experimental>_<data>_<the name of analyze>.xlsx. The pdf and zip files are uniquely identified used the format: <raw>_<exp>_<data>_<the name of analyze>.pdf/zip.

The sampling point is located on the slope of the NW Black Sea continental platform at the exact location described by 43.925.917 N, 30.758.911 E (see Fig. 1 from [2]) and at a water depth of 658 m in the so called anoxic/euxinic environment.

In the coastal area these parameters helped us to identify the sources of OM among the lacustrine/marine algae and land-plant origins (C3 and C4) in the sedimentary remains (see Fig. 2 from [2]) [3,5].

The radiocarbon ages for 25 samples obtained on bulk OM as uncorrected and corrected ages are presented in Table 1 from [2] or like “Experimental_data_14C.xlsx”. The radiocarbon raw data files “raw_exp_data_14C.zip” (.dat files) work with BATS 4.06 data reduction software. The age of the most upper sample was discarded due to the presence of bomb-peak ^14^C. The corrections occur as a result of the reservoir effect of the OM and organic detritus influence by using the values obtained in [4], i.e., 60 as marine reservoir effect and 580 years as detritus organic carbon respectively, cumulating a total R of 640 years for the OM.

**Table 1:** Nitrogen and stable carbon isotopes.

Nr. Crt.	Sample code	Depth (cm)	C/N atomic ratio	δ^13^C_VPDB_ (‰)
1	G2861	0-2	10.55 ± 0.5	-25.3 ± 0.5
2	G2964	2-4	10.60 ± 0.5	
3	G2965	4-6	10.56 ± 0.5	
4	G2966	6-8	10.86 ± 0.5	-24.7 ± 0.5
5	G2755	8-10	11.35 ± 0.5	
6	G2967	10-12	11.02 ± 0.5	-24.4 ± 0.5
7	G2968	12-14	11.01 ± 0.5	
8	G2969	14-16	11.22 ± 0.5	
9	G2970	16-18	10.93 ± 0.5	-24.6 ± 0.5
10	G2756	18-20	11.80 ± 0.5	
11	G2971	20-22	11.42 ± 0.5	
12	G2972	22-24	11.31 ± 0.5	-25.5 ± 0.5
13	G2973	24-26	11.23 ± 0.5	-25.8 ± 0.5
14	G2974	26-28	11.22 ± 0.5	
15	G2757	28-30	12.16 ± 0.5	-24.8 ± 0.5
16	G2975	30-32	11.40 ± 0.5	
17	G2976	32-34	10.26 ± 0.5	-25.1 ± 0.5
18	G2977	34-36	11.07 ± 0.5	
19	G2978	36-38	11.28 ± 0.5	-24.0 ± 0.5
20	G2979	38-40	10,83 ± 0.5	
21	G2758	40-42	11.89 ± 0.5	-24.9 ± 0.5
22	G2980	42-44	10.82 ± 0.5	
23	G2981	44-46	10.93 ± 0.5	
24	G2982	46-48	11.65 ± 0.5	-26.2 ± 0.5
25	G2983	48-50	12.51 ± 0.5	
26	G2759	50-52	14.98 ± 0.5	-23.5 ± 0.5

Marine samples contain two distinct forms of carbon: Organic Carbon (OC) and Inorganic Carbon (IC). These two carbon fractions are presented in Table 2, where the name of the samples, the sampling depth and the amount of total carbon are also found. Total OC (TOC) content can be measured indirectly by the difference between the total carbon content and the inorganic carbon contents. TOC is an important component of sediments that can help distinguish between marine and terrestrial sources of OM, soil quality, sea productivity and allochthonous input indicators.

**Table 2:** Carbon types.

Nr. Crt.	Sample code	Depth (cm)	TC (%)	TOC (%)	IC (%)
1	G2861	1	8.91 ± 0.5	4.61 ± 0.5	4.30 ± 0.5
2	G2964	3	7.95 ± 0.5	3.17 ± 0.5	4.78 ± 0.5
3	G2965	5	7.55 ± 0.5	3.14 ± 0.5	4.41 ± 0.5
4	G2966	7	8.22 ± 0.5	2.95 ± 0.5	5.27 ± 0.5
5	G2755	9	8.67 ± 0.5	3.68 ± 0.5	4.99 ± 0.5
6	G2967	11	9.95 ± 0.5	2.27 ± 0.5	7.47 ± 0.5
7	G2968	13	8.91 ± 0.5	3.74 ± 0.5	5.17 ± 0.5
8	G2969	15	8.66 ± 0.5	3.50 ± 0.5	5.16 ± 0.5
9	G2970	17	9.12 ± 0.5	2.75 ± 0.5	6.37 ± 0.5
10	G2756	19	10.50 ± 0.5	2.55 ± 0.5	7.95 ± 0.5
11	G2971	21	9.37 ± 0.5	2.56 ± 0.5	6.81 ± 0.5
12	G2972	23	8.00 ± 0.5	3.08 ± 0.5	4.92 ± 0.5
13	G2973	25	5.78 ± 0.5	3.35 ± 0.5	2.43 ± 0.5
14	G2974	27	5.89 ± 0.5	2.85 ± 0.5	3.04 ± 0.5
15	G2757	29	7.01 ± 0.5	3.61 ± 0.5	3.40 ± 0.5
16	G2975	31	6.76 ± 0.5	5.01 ± 0.5	1.75 ± 0.5
17	G2976	33	5.78 ± 0.5	4.45 ± 0.5	1.33 ± 0.5
18	G2977	35	6.45 ± 0.5	4.76 ± 0.5	1.49 ± 0.5
19	G2978	37	6.35 ± 0.5	4.79 ± 0.5	1.56 ± 0.5
20	G2979	39	7.13 ± 0.5	5.51 ± 0.5	1.62 ± 0.5
21	G2758	41	8.97 ± 0.5	6.96 ± 0.5	2.09 ± 0.5
22	G2980	43	8.65 ± 0.5	7.22 ± 0.5	1.43 ± 0.5
23	G2981	45	7.99 ± 0.5	6.91 ± 0.5	1.08 ± 0.5
24	G2982	47	9.19 ± 0.5	8.30 ± 0.5	0.89 ± 0.5
25	G2983	49	14.97 ± 0.5	13.60 ± 0.5	1.37 ± 0.5
26	G2759	51	16.89 ± 0.5	15.39 ± 0.5	1.50 ± 0.5

The levels of ^210^Pb and ^137^Cs radioactivity for nine samples, given in Table 3, were used as time-limits for setting the proper radiocarbon age corrections and anchor the age-depth model at the top level. Geochemical evidence of different metallic traces is related to the variable character of the marine/terrigenous influence. These markers which are recorded as depth profiles can be grouped as Ti, K, Fe (allochthonous-terrigenous) and Ca, Sr (autochthonous-biogenic). In this regard, the complete XRF results are presented in "Experimental_data_XRF.xlsx" and the raw data for the XRF measurements is found in "raw_exp_data_XRF.zip". The XRF files (.pdz files) are compatible with Bruker Instrument Tools 1.6.0.110 software.

**Table 3:** ^210^Pb and ^137^Cs radioactivity.

Nr. Crt.	Sample code	Depth (cm)	^210^Pb	^137^Cs
1	G2861	1	1148 ± 221	52 ± 4
2	G2964	3	119 ± 26	1.4 ± 0.5
3	G2965	5	44 ± 20	< 1.1
4	G2966	7	27 ± 13	< 0.9
5	G2755	9	< 131	< 1.0
6	G2756	19	< 90	< 1.1
7	G2757	29	97 ± 13	< 0.9
8	G2758	41	105 ± 50	< 0.9
9	G2759	51	71 ± 47	< 0.9

## 2 Experimental Design, Materials and Methods

### 2.1. ^14^C Dating

A key assumption in the radiocarbon dating consists in removing all the contamination, which might be present in the sample, called ‘pre-treatment’. The pre-treatment normally consists of physical and chemical steps needed to remove the most likely contaminants. In our study, the bulk OM required chemical treatment in order to separate the OC fraction by leaching the IC from the sample. This necessity arises from the simple fact that the IC undergoes different pathways in aquatic systems sedimentation, often presenting higher reservoir effects and therefore different radiocarbon ages compared to the OC. The process involved sample dissolution within a 0.5 M HCl acidic solution for 96 h followed by ultrapure water rinsing, until the pH was neutralized to a value of 5. In the end, the sample was dried at 40° Celsius for 24 h. For the conversion of organic samples to graphite, the samples were combusted and the resulting CO_2_ was subsequently converted to graphite using the AGE III graphitization system [10], which worked in conjunction with a Vario MicroCube elemental analyzer (Elementar, Germany).

For the AMS measurements, all the samples were normalized to the modern radiocarbon level using the international standard Oxalic Acid II [7], while the blank level was estimated using old charcoal of an unknown source. The measurement was performed on IFIN-HH's 1 MV Tandetron^TM^ AMS system. The experimental data obtained for all the samples were corrected for the isotopic fractionation introduced during the sample preparation and spectrometer measurement using AMS δ^13^C. The radiocarbon ages were calculated according to [9].

### 2.2. Carbon Types, Nitrogen and Stable Carbon Isotopes

The TOC content in solids plays an important role in the classification of soils and sediments. TOC is a fast and simple determination method and, at the same time, a robust and statistically reliable method that is based on the homogeneity composition of soils. For the total carbon (TC) determination the weighed solid samples were combusted at 1000°C in a stream of air or oxygen. The CO_2_ generated during combustion is subsequently detected and quantified using a calibration curve. For the IC measurements, a mass of approximately 30 mg of the dried and ground sample was acidified in a solution of 20% phosphoric acid. The acidic solution serves to break down the carbonates present in the sample and, at the same time, form the suspension medium. The measurement was performed on Primacs analyser from Skalar and the resulted CO_2_ was separated and directed into a non-dispersive infrared detection cell for mass determination. The TOC content was further obtained by subtracting the IC content from the TC content (TOC = TC – IC).

The atomic C/N ratio was estimated using Vario MicroCube elemental analyser (Elementar, Germany). Approximately 20 mg of the dried and ground sample is wrapped in a tin foil which is dropped into the combustion column and burned at 900°C in a stream of oxygen, while helium acts as the carrier gas. The excess of oxygen is removed in the reduction column with copper and the combustion gasses (N_2_ and CO_2_) and H_2_O are then separated. The resulting individual gasses in the carrier gas are detected by measuring the thermo-conductivity.

### 2.3. δ ^13^C

The δ^13^C can be used as a biomarker, to distinguish the source of the OM: lacustrine/marine algae or land-plant (C3 and C4). This parameter reflects only to a small extent the water temperature variations, although temperature affects the solubility of CO_2_ into seawater and thus indirectly the δ^13^C composition of phytoplankton and marine carbonates.

For organic δ^13^C measurements, the sediment samples required chemical pretreatment to remove carbonates, due to the fact that IC present in carbonates is more enriched in ^13^C than the OC and could compromise the analysis of OM if it is not correctly eliminated. The process involves the sample dissolution within a 1M HCl for 24 h, after that washed with ultrapure water, dried at 55°C for 48 h and grounded again to obtain a fine powder.

The carbon isotope fingerprint was determined using an isotope ratio mass spectrometer (IRMS) (Delta V Advantage, Thermo Fisher Scientific). The resulting δ ^13^C was normalized to Vienna Pee Dee Belemnite (VPDB) international standard [1], and calibrated against the International Atomic Energy Agency (IAEA) reference material, NBS-22 oil.

### 2.4. X-ray Fluorescence Spectrometry

X-ray fluorescence spectrometry, or XRF, is a non-destructive nuclear analytical technique. It requires minimal sample preparation (in this case, about 0.5 g of dry and homogenous sample powder was pressed into 13 mm diameter pellets by using stainless steel dies and a hydraulic press) and lends itself well to automation. It can detect elements from Na to U in solid, liquid, or powdered samples, from the ppm range up to 100%. Results from this technique can be very reproducible, provided that the sample is introduced in a homogeneous form. In our study, a Bruker Tracer S1 Titan Spectrometer was used, whose beam with energy of 40 keV was passed through an 8 mm collimator before reaching the samples. The spectra were taken using a silicon drift detector (Amptek, USA) positioned backward at 45° with respect to the Rh anode tube.

### 2.5. ^210^Pb and ^137^Cs Radioactivity

A gamma spectrometer with an HPGe semiconductor detector, 30% relative efficiency, model Ortec GMX-30-70-S, equipped with a 0.5 mm Be window was used to measure the levels of the ^137^Cs and ^210^Pb isotopes present in the cores. The presence of ^137^Cs preserved in the sediments is associated with nuclear weapon testing, and its initiation is interpreted to correspond to around 1954. ^210^Pb activity within the sediment can be used to estimate accretion rates. ^210^Pb is a product of the uranium-decay series where ^226^Ra within the crust decays to ^222^Rn. A fraction of the ^222^Rn enters the atmosphere where it decays to ^210^Pb, which quickly precipitates out of the atmosphere, and then is deposited at the surface, and decays with a half-life of 22.3 years.

To reduce the natural background a 10 cm thick lead shield, coated with Sn and Cu foils (1 mm and 1.5 mm, respectively), mounted around the detector was used.

For the measurement, the dried and homogenized samples were introduced in polyethylene containers and sealed for 21 days to prevent the diffusion of ^222^Rn gas and also to establish the secular equilibrium with the short-lived daughter isotopes. Then, the samples were measured for at least 24 h. The total ^210^Pb (E_γ_ = 46.5 keV) and ^137^Cs (E_γ_ = 661.6 keV) from the sediments was measured directly. The supported ^210^Pb was measured indirectly using the daughter isotopes, ^226^Ra and ^214^Bi from ^238^U natural radioactive series, while, the unsupported ^210^Pb was calculated as the difference between total ^210^Pb and supported ^210^Pb according to the constant rate supply model described in [8].

### 2.6. External Data Repository

Sava, Tiberiu Bogdan; Ilie, Maria; Cristea, Gabriela; Ion, Gabriel; Olteanu, Dan; Sava, Gabriela; Mănăilescu, Cristian (2022), “MN183-3 detailed chronology”, Mendeley Data, V1, doi: 10.17632/4b8vhb45z2.1

## CRediT authorship contribution statement

**Maria Ilie:** Methodology, Data curation, Writing – original draft. **Tiberiu Sava:** Conceptualization, Data curation, Writing – original draft. **Gabriela Cristea:** Methodology, Data curation. **Gabriel Ion:** Investigation. **Dan Olteanu:** Methodology, Data curation. **Cristian Mănăilescu:** Methodology, Writing – original draft. **Gabriela Sava:** Methodology, Data curation.

## Declaration of Competing Interest

The authors declare that they have no known competing financial interests or personal relationships that could have appeared to influence the work reported in this paper.

## Data Availability

MN183-3 detailed chronology (Original data) (Mendeley Data).

## References

[1] Brand W.A., Coplen T.B., Vogl J. (2014). Assessment of international reference materials for stable isotope ratio analysis (IUPAC Technical Report). Pure Appl. Chem..

[2] Ilie M., Sava T., Vespremeanu-Stroe A., Duliu O.G., Cristea G., Ion G., Olteanu D., Haliuc A., Mănăilescu C., Sava G. (2022). A detailed chronology of the sedimentation in the Danube abyssal fan records the major episodes of the late-Holocene Black Sea evolution. Quat. Geochronol..

[3] Jasper J.P., Gagosian R.B. (1990). The sources and deposition of organic matter in the late quaternary pygmy Basin, Gulf of Mexico. Geochim. Cosmochim. Acta.

[4] Jones G.A., Gagnon A.R. (1994). Radiocarbon chronology of Black Sea sediments. Deep. Res. Part I.

[5] Meyers P.A. (1994). Preservation of elemental and isotopic source identification of sedimentary organic matter. Chem. Geol..

[6] NIST 1646a, https://www-s.nist.gov/srmors/certificates/1646a.pdf.

[7] NIST SRM 4990C, International standard reference material for contemporary carbon-14. 1983.

[8] Sanchez-Cabeza J.A., Ruiz-Fernandez A.C. (2012). 210Pb sediment radiochronology: an integrated formulation and classification of dating models. Geochimica et Cosmochim. Acta.

[9] Stuiver M., Polach H. (1977). Discussion: reporting of 14C data. Radiocarbon.

[10] Wacker L., Christl M., Synal H.A. (2010). Bats: a new tool for AMS data reduction. Nucl. Instrum. Methods Phys. Res. Sect. B Beam Interact. Mater. Atoms.

[11] Wacker L., Němec M., Bourquin J. (2010). A revolutionary graphitisation system: fully automated, compact and simple. Nucl. Instrum. Methods Phys. Res. Sect. B Beam Interact. Mater. Atoms.

